# Medical Costs of Nontuberculous Mycobacterial Pulmonary Disease, South Korea, 2015–2019

**DOI:** 10.3201/eid3009.231448

**Published:** 2024-09

**Authors:** Shihwan Chang, Sol Kim, Young Ae Kang, Moo Suk Park, Hojoon Sohn, Youngmok Park

**Affiliations:** Yonsei University College of Medicine, Seoul, South Korea (S. Chang, S. Kim, Y.A. Kang, M.S. Park, Y. Park);; Severance Hospital, Seoul (S. Chang, S. Kim, Y.A. Kang, M.S. Park, Y. Park);; Seoul National University College of Medicine, Seoul (H. Sohn);; Seoul National University Institute of Health Policy and Management, Seoul (H. Sohn)

**Keywords:** Nontuberculous mycobacteria, cost of illness, healthcare costs, health expenditures, tuberculosis and other mycobacteria, pulmonary disease, South Korea, bacteria

## Abstract

Nontuberculous mycobacterial pulmonary disease (NTM-PD) prevalence is a rising public health concern. We assessed the long-term healthcare systems perspective of costs incurred by 147 NTM-PD patients at a tertiary hospital in South Korea. Median cumulative total medical cost in managing NTM-PD patients was US $5,044 (interquartile range US $3,586–$9,680) over 49.7 months (interquartile range 33.0–68.2 months) of follow-up. The major cost drivers were diagnostic testing and medication, accounting for 59.6% of total costs. Higher costs were associated with hospitalization for *Mycobacterium abscessus* infection and pulmonary comorbidities. Of the total medical care costs, 50.2% were patient co-payments resulting from limited national health insurance coverage. As South Korea faces significant problems of poverty during old age and increasing NTM-PD prevalence, the financial and socio-economic burden of NTM-PD may become a major public health concern that should be considered with regard to adequate strategies for NTM-PD patients.

Nontuberculous mycobacteria pulmonary disease (NTM-PD) is a chronic respiratory condition of growing concern. Its management is often complicated by multiple biological, clinical, healthcare-associated, and patient factors. Increased NTM infection can be attributed to the widespread presence of NTM in the environment, multiple transmission routes, and insufficient preventive measures (*1*). Diagnosing NTM-PD alone does not require immediate treatment (*2*), often resulting in a period of medical observation before treatment initiation. Eradication of NTM is further challenged by lengthy treatment, lack of effective treatment regimens, antimicrobial resistance, adverse reactions to treatment, and low adherence to therapeutic guidelines (*2*–*5*). Unfavorable treatment outcomes and frequent recurrence necessitate continued observation even after treatment completion (*6*,*7*). By increasing the NTM-PD disease burden (*1*,*3*,*8*,*9*), such issues pose considerable financial strain for healthcare systems and patients.

Among earlier studies evaluating the NTM-PD burden, only a few reviewed the costs associated with NTM-PD (*10*,*11*). A 2017 study in Germany reported a nearly 4-fold increase in the mean direct medical expenditure for patients with NTM-PD compared with those who had never had NTM-PD (*12*). A 2020 study in Canada estimated that NTM-PD management required an annual mean cost of US $11,541 (*13*). Regardless of between-country differences in costs associated with NTM-PD, evidence suggests that NTM-PD poses a substantial cost burden from the healthcare provider perspective (*14*).

To better elucidate the cost burdens associated with NTM-PD, we analyzed the medical costs incurred by patients with NTM-PD in South Korea. Furthermore, we aimed to determine the distribution of costs over the follow-up period and the effects of causative NTM species or pulmonary comorbidities on the costs. Our study was approved by the Institutional Review Board of Severance Hospital (4-2021-1663), with an informed consent waiver considering its retrospective design.

## Methods

**Figure 1:**
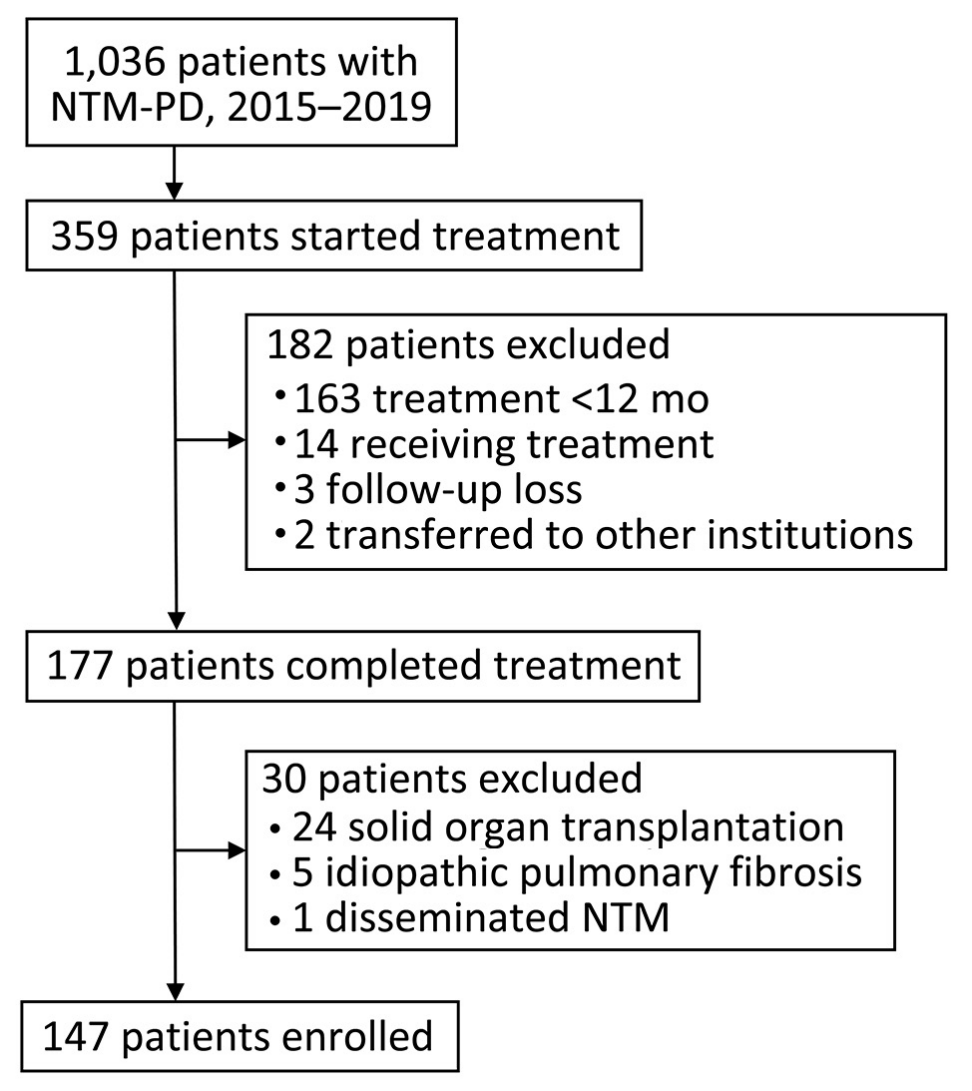
Patient selection for study of medical costs of nontuberculous mycobacterial pulmonary disease, South Korea, 2015–2019. NTM, nontuberculous mycobacteria; PD, pulmonary disease.

We retrospectively reviewed electronic health records (EHRs) and institution billing records (IBRs) of patients who had NTM-PD during 2015–2019 in Severance Hospital, a tertiary referral hospital in South Korea. We restricted the review of medical cost data to patients who had initiated and completed >12 months of NTM-PD treatment before February 2022 (Figure 1). We did not analyze costs incurred by healthcare services provided by or prescribed from institutions other than Severance Hospital.

We extracted patient-level data from the EHR on clinical history, laboratory and imaging tests, prescribed treatment regimens, treatment outcomes, and fee-for-service costs for all relevant medical procedures and care services used by the included patients leading up to February 28, 2022. For costs that could not be ascertained from the IBRs (e.g., medications or health services prescribed from Severance Hospital but received from elsewhere), we referenced the Korean Health Insurance Review and Assessment Service catalog for unit cost/prices (as of January 2019) to calculate estimated total medical costs (*15*). We based the definition of direct medical out-of-pocket costs on patient co-payment amount assessed for each health service used, estimated by the cost ceilings for each item defined by the National Health Insurance Service (NHIS, https://www.nhis.or.kr) (Appendix Table 1).

**Figure 2:**
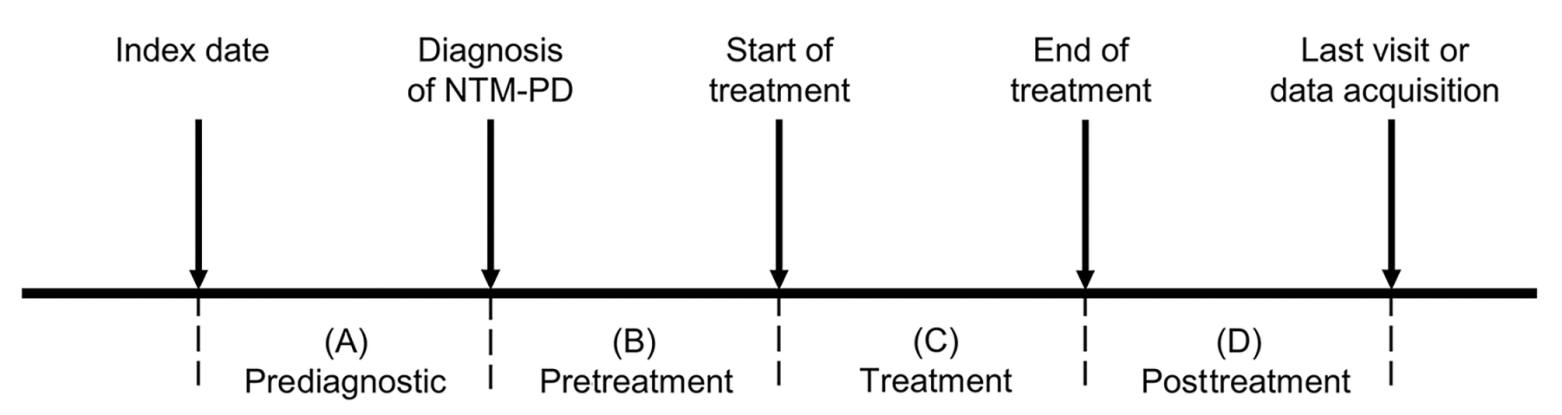
Diagnostic and treatment timeline for study of medical costs of nontuberculous mycobacterial pulmonary disease, South Korea, 2015–2019. The index date was defined as the date of the first pulmonology visit in which the physician initially evaluated the patient for NTM-PD. NTM, nontuberculous mycobacteria; PD, pulmonary disease.

To categorize hospital visits related to NTM-PD management, we divided the follow-up period into 4 periods: prediagnostic, pretreatment, treatment, and post-treatment (Figure 2). We defined the treatment period as the first uninterrupted NTM-PD treatment documented in the EHR.

All costs were assessed from the healthcare system perspective, which includes costs associated with patient co-payments (Table 1). Cumulative per-patient cost was defined as the total costs incurred at all NTM-PD–related visits over the entire follow-up period. Cost assessments were further categorically based on each period of follow-up, type of visit, and medical services. Subgroup analyses were based on NTM species, presence of pulmonary comorbidities, and type of services received (hospital admission, surgical treatment, and management of treatment complications).

**Table 1:** Definitions of visits related to NTM-PD and assessment costs based on category and relevance of treatment used in study of medical costs of NTM-PD, South Korea, 2015–2019*

Variable	Examples
Category	
Medications	All medications, all fees for drug administration (i.e., intravenous access) and dispensing
Diagnostic tests	Acid fast bacilli smear/cultures, NTM identification, NTM drug-susceptibility tests, laboratory tests using patient specimen (i.e., complete blood count, blood chemistry, urinalysis), imaging studies (e.g., computed tomography, radiography), pulmonary function tests, induced sputum, invasive procedures (e.g., bronchoscopy, radiologic intervention)
Clinical services	Doctors’ fees for admission, outpatient visits, and consultation; hospital fees; meals during admission; fees for documentation

Relevance to NTM-PD	
Direct	Antibiotics for NTM-PD treatment, acid fast bacilli smear/cultures, NTM identification, NTM drug-susceptibility tests, laboratory studies for diagnosis and treatment of NTM, surgical management of NTM
Indirect	Medications for symptom control (i.e., cough, sputum, hemoptysis); medications for management of adverse events associated with NTM management; antibiotics and medications for management of pulmonary comorbidities; laboratory studies for all NTM-PD-related visits, unless categorized as direct-related; medical expenses for all NTM-PD-related visits
Unrelated	Costs of management of nonpulmonary comorbidities (i.e., hypertension, diabetes mellitus)

*NTM-PD-related visits were defined as all visits to the pulmonology department and nonpulmonology visits, including outpatient referrals for management of adverse events during NTM-PD treatment, consultations during admissions for NTM-PD management, and visits for surgical management of NTM-PD. Non-NTM-PD–related visits defined as all nonpulmonology visits that do not fit the criteria for an NTM-PD-related visit (i.e., visits for hypertension). NTM, nontuberculous mycobacteria; PD, pulmonary disease.

All costs were assessed as 2019 United States dollars (US $), based on the mean exchange rate for South Korean won and US $ (1,165.69 won/US $1) and adjusted for the medical consumer price index and a 3% discount rate for costs incurred before 2019 (*16*,*17*). For all statistical analyses, we used SPSS Statistics 23 (IBM, https://www.ibm.com) and a 2-tailed significance level of 0.05.

## Results

### Patient Selection and Baseline Characteristics

Of the 1,036 patients with NTM-PD and IBRs for 2015–2019, we included 147 in the final analysis (Figure 1). Median participant age was 61.0 years (interquartile range [IQR] 54.0–66.0 years), 100 (68.0%) participants were female and 47 (32%) were male, and 5 (3.4%) participants had previously received treatment for NTM-PD (Table 2).

**Table 2:** Baseline characteristics of participants in study of medical costs of nontuberculous mycobacterial pulmonary disease, South Korea, 2015–2019*

Variable	Total, n = 147
Age at diagnosis, y, median (IQR)	61.0 (54.0–66.0)
Female	100 (68.0)
Comorbidity	
History of tuberculosis	29 (19.7)
History of NTM treatment	5 (3.4)
Bronchiectasis	36 (24.5)
Chronic obstructive pulmonary disease	21 (14.3)
Asthma	11 (7.5)
Lung cancer	3 (2.0)
History of thoracic operation	12 (8.2)
Hypertension	10 (6.8)
Diabetes mellitus	6 (4.1)
Other malignancy	17 (11.6)
Causative species	
* M. avium* complex	112 (76.2)
* M. abscessus*	15 (10.2)
* M. kansasii*	10 (6.8)
* M. fortuitum*	2 (1.4)
Other†	8 (5.4)
Duration, mo, median (IQR)	
Total duration of follow-up	49.7 (33.0–68.2)
Prediagnostic	0.2 (0.0–2.2)
Pretreatment	3.5 (1.9–8.2)
Treatment	14.8 (13.3–18.4)
Post-treatment	19.5 (9.6–35.6)
No. outpatient visits, median (IQR)	23.0 (18.0–33.0)
No. admissions, n = 71, median (IQR)	1.0 (1.0–2.0)
Length of stay per admission, d, median (IQR)	6.0 (2.0–15.5)
Total length of stay, d, median (IQR)	9.0 (3.0–30.0)
Treatment outcome	
Culture conversion	115 (78.2)
Microbiologic cure	101 (68.7)
Retreatment for recurrence	13 (8.8)
All-cause mortality	6 (4.1)

*Data are presented as no. (%) patients except as indicated. NTM, nontuberculous mycobacteria. †Includes mixed infections.

From the index date, the median follow-up duration of our cohort was 49.7 months (IQR 33.0–68.2 months) and median treatment duration was 14.8 months (IQR 13.3–18.4 months). Over the entire follow-up period, 71 (48.3%) patients were admitted for NTM-PD >1 time.

### Overall Per-Patient Medical Costs

The median per-patient cumulative total cost for NTM-PD treatment was US $5,044 (IQR $3,586–$9,680), or US $1,319 annually (Table 3; Appendix Table 2). Patients incurred a median out-of-pocket cost of US $2,535, which accounted for 50.2% of the total cost. For outpatient visits, patients with NTM-PD incurred a median total cost of US $3,863 (IQR $2,969–$5,333), or US $965 annually per patient. The 71 patients admitted for NTM-PD were hospitalized for a median of 9 days (IQR 3–30 days), incurring a median total admission cost of US $5,620 (IQR $1,296–$9,186), or US $1,146 annually (Appendix Table 3).

**Table 3:** Cost analysis of the treatment of nontuberculous mycobacterial pulmonary disease, South Korea, 2015–2019*

Variable	Median costs (interquartile range)			
	Cumulative†	Out-of-pocket	Annual‡	Per visit§
All visits, n = 147				
Total	5,044 (3,586–9,680)	2,535 (1,779–4,087)	1,319 (845–2,478)	NA
Nonbenefit	115 (13–431)	115 (13–497)	28 (3–109)	NA
Medication	1,197 (656–2,728)	362 (214–872)	296 (178–676)	NA
Diagnostic tests	3,006 (2,134–4,511)	1,536 (1,152–2,332)	701(536– 1,199)	NA
Clinical services	616 (416–1,749)	426 (312–839)	140 (106–481)	NA

Outpatient visits*, *n = 147				
Total	3,863 (2,969–5,333)	2,124 (1,476–2,702)	965 (753–1,370)	163 (134–198)
Nonbenefit	35 (7–115)	35 (7–115)	8 (2–32)	1 (0–5)
Medication	1,050 (584–1,904)	347 (178–664)	247 (159–466)	40 (27–70)
Diagnostic tests	2,282 (1,806–2,997)	1,317 (991–1,772)	598 (433–753)	99 (78–121)
Clinical services	448 (347–597)	368 (272–474)	110 (87–137)	19 (17–20)

Admissions, n = 71				
Total	5,620 (1,296–9,186)	1,494 (506–3,340)	1,146 (291–2,983)	3,493 (1,011–6,141)
Nonbenefit	326 (107–887)	326 (107–887)	65 (24–224)	212 (103–452)
Medication	464 (31–1,773)	94 (11–412)	110 (8–442)	275 (26–900)
Diagnostic tests	1,590 (734–4,405)	498 (233–1,785)	317 (182–1,269)	1,243 (653–2,045)
Clinical services	1,470 (289–4,118)	348 (72–1,000)	359 (66–1,382)	1,111 (200–2,634)

*Currency in US $, rounded to the nearest dollar. NA, not applicable. †All costs pertaining to each category of visit. ‡Cumulative cost divided by the duration of total follow-up in years. §Cumulative cost for outpatients divided by the number of outpatient visits. ¶Cumulative cost of admission divided by the number of admissions.

Patients with any history of hospitalization for NTM-PD–related treatment (median US $9,429) incurred a median total cost 2.5 times greater than those without (median US $3,773) (Appendix Table 4). However, costs for outpatient visits did not differ between both groups, suggesting that hospitalization costs were the main driver of increased medical cost for this patient group.

**Figure 3:**
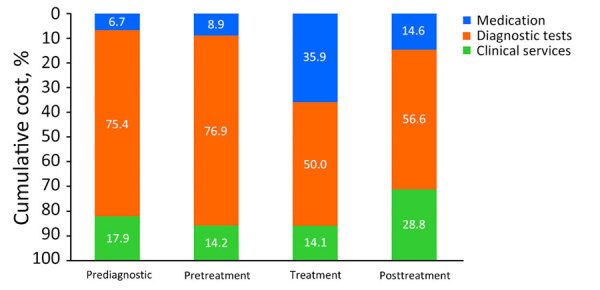
Cost proportion analysis by follow-up period, in study of medical costs of nontuberculous mycobacterial pulmonary disease, South Korea, 2015–2019. Cumulative cost was analyzed by each follow-up period. Numbers within bars represent the percentage of each cost category.

The median cumulative cost during the overall follow-up period was US $5,470 (IQR $3,613–$9,914) (Table 4; Figure 3; Appendix Table 5). The median per-patient cost was highest during the treatment period (US $2,108), followed by the pretreatment (US $616) and post-treatment (US $451) periods. Costs per patient were lowest for the prediagnostic period (US $425). Medical costs sharply increased approximately 6 months before treatment initiation, peaking during the first 3 months. Costs gradually decreased approximately 1 year after treatment initiation (Appendix Figure 1).

**Table 4:** Cumulative cost for each category and proportions of out-of-pocket cost in study of medical costs of nontuberculous mycobacterial pulmonary disease, South Korea, 2015–2019*

Period	Median cost, US$ (IQR)	% Out-of-pocket
Overall follow-up		
Total	5,470 (3,613–9,914)	47.4
Medication	1,171 (550–2,509)	29.6
Diagnostic tests	3,093 (2,134–4,597)	49.9
Clinical services	633 (435–1,749)	75.7
Prediagnostic		
Total	425 (255–1,052)	60.2
Medication	10 (0–77)	30.0
Diagnostic tests	393 (231–766)	56.2
Clinical services	44 (24–99)	86.4
Pretreatment		
Total	616 (399–1,079)	58.3
Medication	18 (0–74)	33.3
Diagnostic tests	497 (279–864)	60.0
Clinical services	65 (40–111)	78.5
Treatment		
Total	2,108 (1,660–3,590)	48.8
Medication	814 (512–1,362)	31.0
Diagnostic tests	1,051 (837–1,726)	56.7
Clinical services	209 (180–303)	84.7
Post-treatment		
Total	451 (138–1,363)	63.6
Medication	7 (0–220)	28.6
Diagnostic tests	295 (94–902)	51.2
Clinical services	89 (42–229)	60.7

*Data are for 87 patients with data for all 4 periods of follow-up.

By types of medical services, costs for diagnostic tests shared the highest cumulative (59.6%, US $3,006), outpatient (59.1%, US $2,282), and admission (54.3%, US $1,590) costs over the entire follow-up period (Table 3). Diagnostic tests consistently accounted for the highest costs in any follow-up period (Table 4, Figure 3). Medication costs accounted for the second largest proportion of the cumulative costs for the entire follow-up period (23.7%, US $1,197) and for those incurred only during treatment (38.6%, US $814) but not in all other periods.

### Subgroup Analyses and Patient-Level Cost Drivers 

When comparing costs by the 2 major NTM species, the overall duration of follow-up was longer for the 112 patients treated for *M. avium* complex (MAC) infection (median 51.4 months) than for the 15 treated for *M. abscessus* infection (median 33.1 months); p = 0.006). All 15 patients with *M. abscessus* infection had been admitted for NTM-PD treatment, experiencing >2 hospitalizations that resulted in total stays of 33 days (IQR 28–62 days). Nearly 40.2% of patients with MAC infection were admitted for a median duration of 5 days (IQR 2–10 days) (Appendix Table 6). Likewise, median medical costs incurred by patients with *M. abscessus* infection (US $19,190) were higher than those incurred by patients with MAC infection (US $4,557; p<0.001) (Table 5; Appendix Tables 7, 8); the main driver of the cost difference between the groups was admission costs.

**Table 5:** Analysis of cumulative cost based on causative species in study of medical costs of nontuberculous mycobacterial pulmonary disease, South Korea, 2015–2019*

Variable	Costs, median (interquartile range)		
	*Mycobacterium avium* complex, n = 112	*M. abscessus*, n = 15	p value
All visits			
Total	4,557 (3,334–7,086)	19,190 (9,914–26,219)	<0.001
Medication	1,069 (614–1,799)	2,767 (2,441–4,225)	<0.001
Diagnostic tests	2,830 (2,078–4,055)	5,726 (3,334–11,190)	<0.001
Clinical services	541 (377–930)	6,093 (4,263–11,972)	<0.001
Outpatient visits			
Total	3,859 (2,960–5,124)	3,300 (3,016–4,278)	0.492
Medication	976 (543–1,645)	983 (738–1,466)	0.823
Diagnostic tests	2,292 (1,805–3,060)	1,863 (1,650–2,480)	0.110
Clinical services	444 (347–564)	506 (318–643)	0.399
Admissions†			
Total	1,710 (933–7,747)	14,197 (6,711–22,521)	<0.001
Medication	61 (20–516)	1,784 (1,456–3,153)	<0.001
Diagnostic tests	998 (653–4,793)	3,093 (1,590–9,867)	0.024
Clinical services	556 (147–1,863)	5,639 (3,847–11,439)	<0.001

*Currency in US $, rounded to the nearest dollar. †*M. avium* complex n = 45.

Regarding patient-level factors, the number of outpatient and admission visits, total length of stay, and death associated with *M. abscessus* infection (compared with MAC infection) were associated with increased total medical care costs for NTM-PD (Appendix Table 9). Multiple linear regression results revealed that the number of admissions and *M. abscessus* infections (compared with MAC infections) were positively associated with the total medical cost (i.e., more admissions and *M. abscessus* infections increased costs), whereas culture conversion was associated with decreased total cost (Table 6).

**Table 6:** Multiple linear regression analysis of factors driving cumulative cost in study of medical costs of nontuberculous mycobacterial pulmonary disease, South Korea, 2015–2019*

Model no., variable(s)	B	SE	β	t	p value	R^2^	Adjusted R^2^
1							
No. admissions	6058.547	627.484	0.785	9.655	<0.001	0.616	0.610
2						0.739	0.730
No. admissions	5503.292	532.913	0.713	10.327	<0.001		
Causative species: MAC = 0, *M. abscessus* = 1	7921.642	1530.022	0.358	5.177	<0.001		
3						0.758	0.746
No. admissions	5379.334	520.642	0.697	10.332	<0.001		
Causative species: MAC = 0, *M. abscessus* = 1	7445.211	1502.260	0.336	4.956	<0.001		
Culture conversion	−3082.792	1455.967	−0.142	−2.117	0.039		

*N = 147 patients. Variables include age, sex, history of tuberculosis, history of nontuberculous mycobacteria treatment, bronchiectasis, asthma, chronic obstructive pulmonary disease, lung cancer, causative species (MAC vs. *M. abscessus*), total follow-up duration, treatment duration, number of outpatient visits, number of admissions, culture conversion, microbiologic cure, retreatment for recurrence, and death from any cause. MAC, *Mycobacterium avium* complex; NA, not applicable.

### Out-of-Pocket Costs

Patients incurred a median out-of-pocket cost of US $2,535 (Table 3), accounting for 50.2% of the cumulative cost. The co-payment for outpatient visits and admissions was 55% (median US $2,124) and 26.6% (median US $1,494) of the cumulative costs for the respective visits. Similar to the patterns observed for cumulative costs, out-of-pocket costs over the follow-up period indicated that the highest co-payment was observed for the treatment period (US $1,029), followed by the pretreatment (US $359) and post-treatment (US $287) periods (Table 4; Figure 3).

## Discussion

Our report of comprehensive and detailed NTM-PD medical care costs in South Korea indicates that each patient with NTM-PD incurred a total cost of US $5,044 over a median follow-up period of 49.7 months, which translates to a median annual cost of US $1,319. Restricting analysis to the 87 patients with complete follow-up data brings the total median cost slightly higher, to US $5,470. Our analyses revealed that most NTM-PD–associated medical services were not fully covered through the Korean NHIS, resulting in a median out-of-pocket cost accounting for 50.2% (US $2,535) of the total care costs. Most (66.4%) of the total NTM-PD medical costs were incurred between 6 months before and 1 year after treatment initiation. The largest share of the total medical costs during the entire follow-up period was costs associated with diagnostic tests. Medical costs were higher for patients with *M. abscessus* infection and those with pulmonary comorbidities because of more frequent hospital visits and more extended hospitalizations.

In earlier studies evaluating medical costs of pulmonary infections in South Korea, medical costs for NTM-PD were considerably higher than those for asthma (US $267, reported in 2017) and drug-susceptible tuberculosis (US $754, reported in 2022) and were comparable to the costs for treating and managing tuberculosis-destroyed lungs (US $1,838, reported in 2019) (*18*–*20*). Furthermore, a recent 2023 study conducted by our team using the NHIS database reported that patients with NTM infection incurred at least 1.5 times higher annual medical costs than the healthy control population (a total annual medical cost of US $2,279.99 vs. US $1,496.26) (*21*). In addition, patients with NTM-PD incurred large co-payments or direct out-of-pocket costs for medical services not covered through the NHIS. Those costs comprised >50% of the total medical costs, suggesting that long-term disease burden directly translates to a considerable cost burden for patients with NTM-PD compared with other chronic respiratory conditions (*22*,*23*).

We report the long-term costs associated with NTM-PD and the cost drivers by different phases of disease management, types of medical services, and parts of the medical costs borne by patients. Costs start to increase approximately 6 months before treatment initiation, peak during the first quarter of treatment, and then decrease after approximately 1 year of treatment (Appendix Figure 1). That period, which roughly coincides with the pretreatment and treatment periods (Table 2), accounts for 66.4% of the total cost (Appendix Figure 2). A combination of factors may explain the cost concentration observed throughout that period, when intensive laboratory studies are needed to decide when to initiate treatment and monitor treatment outcomes as well as treatment and management of associated adverse events. Previous studies have demonstrated that the economic burden is greater during the early follow-up phases. In a recent study that compared healthcare expenditures among patients with NTM infection and matched controls, medical costs were the highest 6 months before NTM-PD diagnosis (*21*). Similar studies conducted in Germany and the United States reported that annual overall healthcare costs were highest during the first year after NTM-PD diagnosis (*12*,*24*). Another study conducted in Canada demonstrated that costs attributable to NTM-PD during the acute-care phase (i.e., the first 150 days after the first hospital visit associated with NTM-PD) were greater than those associated with subsequent care phases (*13*).

Earlier studies have identified hospitalization costs as the main cost driver of NTM-PD management (*12*,*13*,*24*). Among our study population, 71 (48.3%) patients were admitted for NTM-PD–related issues throughout the follow-up period. Admission costs accounted for ≈59.6% of the total costs incurred by those patients, consistent with earlier findings reporting hospitalization costs accounting for as much as 69% of the total medical costs for NTM-PD (*14*). In our study, the main cost drivers for hospital admissions (54.3%) and the entire NTM-PD management (59.6%) were diagnostic tests (Table 3). The second largest cost driver, medication costs (23.7% of the total cost), was concentrated in the treatment period (38.4% of the costs during treatment) (Table 3; Appendix Figure 2). Our estimates were higher than those in France (6%), Germany (9%–21.8%), and the United Kingdom (12%) but lower than those in Canada (6%–70%), which may reflect differences in clinical practice and patient characteristics across countries (*11*,*12*,*14*). Furthermore, a relatively lower proportion of medication costs for managing NTM-PD in South Korea may be attributed to the government-negotiated drug costs for those approved for reimbursement schemes by the NHIS, which are generally lower than the market prices in other high-income settings (*25*).

Clinically challenging patient subgroups, such as those with *M. abscessus* infections (*2*,*26*) or comorbidities, probably require more healthcare resources and incur more costs for disease management (*10*,*11*). Our study directly assessed the increased costs and cost drivers associated with managing more difficult subgroups of patients with NTM-PD in South Korea. Patients with the *M. abscessus* subtype incurred costs 4.2 times higher than those infected with MAC, which was almost exclusively because of the increased costs associated with longer and more frequent hospitalizations for patients with *M. abscessus* infection (Table 5; Appendix Table 6). Moreover, patients with pulmonary comorbidities bore >67.4% more costs to treat and manage NTM-PD than those without comorbidities (Appendix Tables 10–12). NTM-PD patients with pulmonary comorbidities incurred approximately twice as much medication cost and a 50% increase in diagnostic test costs. When comparing the cost driver by types of clinical visits, we found that most of the increased medical costs were attributable to hospitalization costs.

Our cost estimates of NTM-PD management in South Korea were considerably lower than those in Germany and Canada. In 2011, Leber et al. estimated an annual cost of approximately CA $6,000 required to treat NTM-PD in Canada (*11*). In 2017, researchers in Germany compared the healthcare costs of patients with NTM-PD with those of uninfected controls and reported an attributable direct annual cost associated with NTM-PD of €9,093.20 (*12*). Our estimate of US $1,319 as the annual cost of managing NTM-PD is substantially lower in absolute terms; the large difference in medical costs for NTM-PD may be attributable to lower fee-for-service costs for medical care in South Korea than in Germany or Canada. According to a 2021 health report of the Organisation for Economic Cooperation and Development (OECD, https://www.oecd.org), per-capita healthcare expenditure in Korea (US $3,406) is approximately half of that in Canada (US $5,370) and Germany (US $6,518); however, healthcare use (e.g., annual consultations per person) far exceeds (17.2 in 2019 for South Korea) that of Germany (9.8) and Canada (6.6) (*23*). Those discrepancies may result from considerable differences in medical fee schedules across those countries. If medical service fees in South Korea are equivalent to those of Canada and Germany, costs associated with NTM-PD management in South Korea would be equivalent to, if not higher than, those of Canada and Germany.

Although our study did not assess patient perspective costs, we were able to assess patient co-payment costs associated with all medical services used through NTM-PD management. Patients incurring higher medical costs experienced higher co-payment and out-of-pocket costs for their illness management. Except for patients with *M. abscessus* infection, patients in our cohort paid approximately more than half of the cumulative total medical care costs incurred during their disease management at our institution alone, which represents a much larger share (in terms of co-payment) of medical costs relative to the general South Korea population average (30%) and >3 times the share of costs reported from Canada (15%) and Germany (13%) (*23*).

Given the limited scope of our data, we were not able to ascertain cost burdens associated with nonmedical direct and indirect costs. As such, the direct patient out-of-pocket medical care costs assessed represent only ≈2% of the South Korea Gross Domestic Product per capita. However, South Korea faces substantial problems with poverty during old age, which affects 43.8% of the elderly population according to the OECD. That proportion is the highest in the OECD rankings and 3 times greater than the OECD average of 13.5% (*27*). NTM-PD patients in our study and South Korea in general are elderly (*8*,*21*,*28*). Given those considerations, we suspect that NTM-PD may impose major socio-economic burdens on the patients, which may be much more significant for particular subgroups (e.g., those with multiple hospitalizations or *M. abscessus* infections and those who remain culture positive despite treatment). More research is needed to better understand the socio-economic consequences of NTM-PD (including the level of catastrophic costs experienced by the patient and their household because of NTM-PD) and guide the development of relevant policy measures.

Among the interpretation and generalizability limitations of our study, our study cohort was derived from a single institution. Thus, our cost estimates may not fully represent the general medical costs of NTM-PD management across South Korea. It is possible that NTM-PD patients included in our sample may have sought care for NTM-PD at other institutions before and during follow-up visits to Severance Hospital, which may result in higher healthcare systems perspective per-patient costs. However, annual per-patient NTM management costs reported from an earlier study in South Korea that used the national health insurance claims database (healthcare systems perspective) (US $1,197.75/y) were equivalent to (or slightly lower than) our cost estimates (US $1,319/y) (*21*). Although we are not able to directly compare the 2 studies by using patient-level data, similarities in the cost estimates suggest that many NTM patients may be managed at a single institution and that our estimates closely represent those of general NTM patients in South Korea. Although it is difficult to ascertain and attribute differences in NTM-PD medical costs attributed to patients in South Korea compared with those in other countries, similarities in the overall trends (key cost drivers by types of healthcare use and types of diseases) suggest that the magnitude of our estimates largely represents differences in clinical practices and medical service costs across other countries with similar estimates. Second, we were limited in assessing any additional NTM-PD–related costs that patients and the healthcare systems may have incurred, such as nonmedical costs and productivity loss. Therefore, our cost estimates may underestimate true costs for NTM-PD management. Data from patient cost survey studies (to ascertain short- and long-term patient costs) and large panel data comprising multi-institutional cohorts with medical insurance claims information may provide a complete disease and financial burden of NTM-PD. Last, although we compared the costs associated with NTM-PD with those associated with other respiratory conditions, lack of an adequate control group with no history of NTM-PD limited our assessment of the relative disease and financial burden of NTM-PD.

In conclusion, our comprehensive, long-term assessment of medical costs associated with NTM-PD management demonstrates that NTM-PD poses a substantial disease burden on and financial costs to healthcare services and patients. Our findings suggest that the cost burden is considerably higher for patients with *M. abscessus* infection or pulmonary comorbidities, which is attributable to the longer duration of disease management and more frequent and longer hospitalization required. In addition, unlike patients with other respiratory infection–related illnesses, patients with NTM-PD bear much larger direct out-of-pocket costs that account for approximately 50% of direct medical costs. Although additional evidence from more complete data sources may be needed to fully ascertain long-term disease burden and costs, our study findings provide initial evidence that can be used for developing policies to support patients with NTM-PD and alleviate financial burdens during their disease management.

Appendix Additional information for study of medical costs of nontuberculous mycobacterial pulmonary disease, South Korea, 2015–2019.
