# Supplementary material for: Medical Costs of Nontuberculous Mycobacterial Pulmonary Disease, South Korea, 2015–2019
Source: Emerg Infect Dis. 2024 Sep;30(9):1841–9. doi: 10.3201/eid3009.231448 (PMC11346975; doi:10.3201/eid3009.231448)
Supplement: Appendix — Additional information for study of medical costs of nontuberculous mycobacterial pulmonary disease, South Korea, 2015–2019. [file 23-1448-Techapp-s1.pdf]

Article DOI: https://doi.org/10.3201/eid3009.231448

EID cannot ensure accessibility for supplementary materials supplied by authors. Readers who have difficulty accessing supplementary content should contact the authors for assistance.

Medical Costs of Nontuberculous Mycobacterial Pulmonary Disease, South Korea, 2015–2019

Appendix

Detailed Methods

Study population and case definitions

We retrospectively reviewed electronic health records (EHR) and institutional billings records of patients diagnosed with NTM-PD from 2015 to 2019 in Severance Hospital, South Korea. From the EHR, we first selected patients who satisfied the diagnostic criteria for NTM-PD from the 2007 clinical guidelines (*1*). To assess the long-term medical care cost associated with NTM-PD disease, we restricted the review of medical costs data for those patients who had initiated and completed at least 12 months of NTM-PD treatment before February 2022. We therefore excluded patients falling into following categories: 1) patients with record of NTM-PD treatment for less than 12 months (or whose treatment did not terminate before February 2022), 2) who were loss to follow-up or transferred out, 3) patients with idiopathic pulmonary fibrosis, disseminated NTM disease, or a history of solid organ transplantation.

For NTM species classification, *M. avium* and *M. intracellulare* were grouped as *M. avium* complex (MAC) and *M. abscessus* subspecies *abscessus* and *M. massilliense* subspecies *massilliense* were grouped as MAB. To define treatment outcomes, we used the definitions according to the NTM Network European Trials group consensus statement (*2*). We considered patients to have culture-converted when there was a minimum of three consecutive negative mycobacterial cultures reported during the patient's NTM-PD treatment, with at least 4 weeks between each culture result. We considered microbiologic cure for those patients with multiple consecutive culture-negative results for the causative species post-culture conversion up to the

point of treatment termination. Recurrence was defined as the re-emergence of at least two positive cultures of the causative species after the termination of treatment.

Data acquisition of patients and medical costs

From the EHR, we extracted patient-level data on clinical history, laboratory and imaging tests, prescribed treatment regimens, treatment outcomes, and fee-for-service costs for all relevant medical procedures and care services used by the patients included in our study leading up to 28 February 2022. All cost estimates included overhead, and administrative costs allocated to each health service provided at Severance Hospital. For health services used by the NTM-PD patients that are not covered by the National Health Insurance Service (NHIS) plan, we accounted these costs as part of the total health services costs, but tracked separately as non-benefits costs to assess these costs as part of patients out-of-pocket (may be reimbursed through patient's private healthcare insurance plan) costs.

For costs that could not be ascertained from the institution billings records (e.g., medications or health services prescribed from Severance Hospital, but received out-of-the institution), we referenced the Korean Health Insurance Review and Assessment Service (KHIRA) catalog for unit cost/prices (as of January 2019) to calculate estimated total medical costs (multiplied by actual prescription amount) (3). Out-of-pocket costs for these costs were estimated by the cost ceilings defined for each item by the NHIS (Appendix Table 1).

Classification, calculation, and analyses of per-patient medical costs

To categorize hospital visits related to NTM-PD management, we reviewed the EHR for all visits made by the patient after the index date, defined as the first pulmonology visit in which the physician initially evaluated for NTM-PD. To ascertain costs by different periods of NTM-PD care-seeking, we categorized medical service use and costs by four distinct periods: (1) pre-diagnostic, (2) pre-treatment, (3) treatment, and (4) post-treatment follow-up (Figure 2a). The treatment period was defined as the first period of uninterrupted NTM-PD treatment documented in the EHR.

Table 1 presents the criteria used to categorize the hospital visits and medical services. Medical care visits were categorized as either outpatient visits or admission (includes emergency department visits). NTM-PD-related visits included all visits to the pulmonary care, and visits outside of the pulmonary care for referrals made as part the management of NTM treatment

related adverse events, consultations during NTM-PD-related admissions, or hospitalizations for lung resection performed as part of the strategy for NTM-PD treatment. We excluded health service uses that were associated with managing non-pulmonary comorbidities that were not relevant to NTM-PD disease management in our analysis.

To determine the inclusion and exclusion criteria for medical costs directly and indirectly associated with the NTM-PD treatment and management, we decided based on medical procedures and services with health services codes referencing 1) NTM-PD as primary disease and 2) pulmonary and non-pulmonary comorbidities with reference to NTM-PD (i.e., associated with NTM-PD and treatment). For NTM-PD patients with pulmonary comorbidity, we included health service use that may require more close monitoring during NTM-PD management, which included asthma, chronic obstructive pulmonary disease (COPD), bronchiectasis, and lung cancer. For non-pulmonary comorbidity related health service use, we considered those related to any underlying extrapulmonary conditions, such as hypertension or diabetes mellitus. For surgical interventions, we only considered as costs relevant for NTM-PD if lung resection operations were specifically indicated for NTM-PD treatment.

Costs were further categorized into three types of medical services: medication, diagnostic tests, clinical services. Medication costs included costs for all prescribed drugs and pharmacy fees. Costs for diagnostic tests were assessed based on actual prescription of tests services used by the patient that was related to NTM-PD treatment and management. These included blood biochemistry tests, microbiologic tests, imaging, and invasive procedures, such as surgery, biopsy, and bronchoscopy. Clinical services costs included doctor-visit fees, documentation, and admission fees, which included meal plan costs for overnight admissions.

Assessment of the outcomes and data analysis

Cumulative per-patient cost was defined as the total amount of costs incurred in all NTM-PD-related visits over the entire follow-up period. Costs were further categorically assessed based on each period of follow-up, types of visits and medical services. Annual cost for each item was calculated by dividing total cost by the duration of the corresponding period of follow-up. Out-of-pocket cost was calculated for each medical service items described above and presented as total sum and proportion of the total medical costs to determine the financial burden for the patients. Subgroup analyses were performed based on NTM species, the presence of

pulmonary comorbidities, and the type of services received (hospital admission, surgical treatment, and management of treatment complications).

All costs were assessed as 2019 United States Dollar (US $) based on the mean exchange rate for Korean Won (KRW) and US $ (1165.69 KRW per one US $ in 2019) and adjusted for consumer price index and 3% discount rate for cost incurred in prior years to 2019. Mann–Whitney U test was used to compare median values. Student's t-test was used to compare mean values. Fisher exact test and chi-square tests were used to compare the proportions of costs. To assess the patient-level cost drivers, we performed simple and multiple linear regression analysis (stepwise), using the following variables: age, sex, history of NTM treatment, presence of pulmonary comorbidities, history of tuberculosis (TB), causative species (MAC versus MAB), total duration of follow-up, treatment duration, number of outpatient visits, number of admissions, culture conversion, microbiologic cure, re-treatment for recurrence, and death from any cause. For all analyses, a two-tailed significance level of 0.05 was used. All analyses were performed using SPSS software version 23 (IBM Corporation, Armonk, New York, USA).

Our study was approved by the Institutional Review Board of Severance Hospital (4–2021–1663), with an informed consent waiver considering its retrospective design.

Results

Subgroup analysis: patients with pulmonary comorbidities

There were 63 patients in our cohort with pulmonary comorbidities, namely, asthma, bronchiectasis, COPD, and lung cancer. Patients with pulmonary comorbidities (n = 63) had longer duration of follow-up (median 55.2 months versus 45.8 months, $p = 0.032$) and more outpatient visits (median 27.0 visits versus 21.0 visits, $p = 0.039$) than those without comorbidities (n = 84) (Appendix Table 10). Subsequently, higher costs were incurred by patients with pulmonary comorbidities (cumulative US $6,999) than those without (cumulative US $4,182), regardless of visit types (Appendix Table 11). However, statistical significance in these cost differences were observed for cumulative ($p < 0.001$) and outpatient visits ($p < 0.004$), but not for admissions.

Out-of-pocket costs by subgroup

When compared by causative species, MAB patients were charged more as co-payment than MAC patients (MAB US $5,765 versus MAC US $2,328), even with the lower proportion of co-payment observed in MAB patients. The observed differences in co-payments are driven primary by the higher admission costs seen in MAB patients. (Appendix Table 13). Patients with comorbidities (US $3,156) were charged more co-payment than those without comorbidities (US $2,209, Appendix Table 14). The difference mainly reflects the cost for managing associated comorbidities (Appendix Table 10).

Surgery for NTM-PD, Non-pulmonary visits that were NTM-PD-related.

A total of five patients underwent surgery for NTM-PD treatment (Appendix Table 15), accounting for 47.3% (median US $9,077, IQR 8,566–19,866) of the entire cost of NTM-PD treatment in this patient group (median US $19,190, IQR 14,264–94,780).

81 patients (55.1%) had NTM-PD-related clinical visits that were non-pulmonary visits (Appendix Table 16). Otorhinolaryngology visits occurred in 28 patients (19.0%), primarily to screen for hearing loss associated with intravenous amikacin use, accounting for a median cumulative cost of US $72 per-patient. Twenty-one patients (14.3%) had experienced visual disturbances associated with ethambutol use and required visits to the ophthalmology clinic. These visits accounted for US $147 per-patient. Both of the non-pulmonary clinical visits/consultations accounted for a small fraction (0.5% for otorhinolaryngology and 3.0% for ophthalmology) of the total per-patient medical costs for NTM-PD treatment.

Appendix References

1. Griffith DE, Aksamit T, Brown-Elliott BA, Catanzaro A, Daley C, Gordin F, et al.; ATS Mycobacterial Diseases Subcommittee; American Thoracic Society; Infectious Disease Society of America. An official ATS/IDSA statement: diagnosis, treatment, and prevention of nontuberculous mycobacterial diseases. Am J Respir Crit Care Med. 2007;175:367–416. PubMed https://doi.org/10.1164/rccm.200604-571ST

2. van Ingen J, Aksamit T, Andrejak C, Böttger EC, Cambau E, Daley CL, et al.; for NTM-NET. Treatment outcome definitions in nontuberculous mycobacterial pulmonary disease: an NTM-NET consensus statement. Eur Respir J. 2018;51:1800170. PubMed https://doi.org/10.1183/13993003.00170-2018

3. Health Insurance Review and Assessment Service. Table of Reimbursed Drugs and Price Ceilings. May 2022 [cited 2022 May]. https://www.hira.or.kr/bbsDummy.do?pgmid=HIRAA030014050000

Appendix Table 1. Percentage of co-payment for items covered by the national health insurance system*

Type of visit	Items	Co-payment
Outpatient (tertiary care)	All items, other than exceptions below	60%
	Selective benefits[#]	30–90%
	Doctor's fees	100%
Out-of-institution pharmacy	All items	30%
Admission (tertiary care)	All items, other than exceptions below	20%
	Selective benefits[#]	30–90%
	Meal costs	50%
	Room charges	10–100%

[#]Proportions of co-payment for these items are determined on an item-by-item basis, by national health insurance system.

Appendix Table 2. Cost analysis for the treatment of nontuberculous mycobacterial pulmonary disease, reported in mean and standard error*

	Cumulative cost[#]		Out-of-pocket cost		Annual cost[¶]		Per visit/admission	
Variable	Mean	95% CI	Mean	95% CI	Mean	95% CI	Mean	95% CI
All visits (n = 147)								
Total	8,991	6,603–11,379	3,647	3,016–4,277	2,339	1,760–2,918		
Non-benefit	606	328–885	610	332–889	171	80–262		
Medication	2,258	1,721–2,796	698	528–868	574	430–718		
Diagnostic tests	4,542	3,412–5,671	1,947	1,689–2,205	1,158	909–1,407		
Clinical services	2,191	1,364–3,018	1,002	691–1,312	607	385–828		
Outpatient visits (n = 147)	Cumulative cost[#]		Out-of-pocket cost		Annual cost[¶]		Per visit[+]	
	Mean	95% CI	Mean	95% CI	Mean	95% CI	Mean	95% CI
Total	4,528	4,126–4,929	2,261	2,080–2,443	1,124	1,030–1,217	175	164–186
Non-benefit	90	68–112	92	70–114	26	17–34	3	3–4
Medication	1,559	1,306–1,813	512	431–592	377	320–433	58	50–65
Diagnostic tests	2,486	2,317–2,655	1,368	1,263–1,473	625	580–670	99	93–104
Clinical services	483	450–516	381	353–410	122	111–133	18	18–19
Admission (n = 71)	Cumulative cost[#]		Out-of-pocket cost		Annual cost[¶]		Per admission[§]	
	Mean	95% CI	Mean	95% CI	Mean	95% CI	Mean	95% CI
Total	9,241	4,739–13,743	2,868	1,781–3,955	2,516	1,444–3,588	4,164	3,206–5,122
Non-benefit	1,069	524–1,613	1,073	529–1,617	300	120–480	547	283–810
Medication	1,448	587–2,309	386	119–654	409	167–651	592	407–776
Diagnostic tests	4,257	2,078–6,436	1,198	781–1,614	1,103	634–1,572	1,928	1,405–2,452
Clinical services	3,536	1,955–5,118	1,284	696–1,872	1,005	588–1,421	1,645	1,230–2,060

*Currency in USD, rounded to the nearest dollar.
#Includes all the cost pertaining to each category.
¶Cumulative cost divided by the duration of total follow-up in years.
+Cumulative cost for outpatient divided by the number of outpatient visits.: confidence interval.

Appendix Table 3. Expanded cost analysis for the treatment of nontuberculous mycobacterial pulmonary disease*

	Cumulative cost[#]		Out-of-pocket cost		Annual cost[¶]		Per visit/admission	
Variable	Median	IQR	Median	IQR	Median	IQR	Mean	95% CI
All visits (n = 147)								
Total	5,044	3,586–9,680	2,535	1,779–4,087	1,319	845–2,478		
Non-benefit	115	13–431	115	13–497	28	3–109		
Direct-related	2,361	1,734–3,258	1,044	731–1,444	562	409–893		
Indirect-related	2,546	1,476–6,070	1,369	809–2,257	566	380–1,567		
Unrelated	30	1–212	13	0–90	9	0–41		
Medication	1,197	656–2,728	362	214–872	296	178–676		
Direct-related	748	497–1,367	225	149–410	189	125–355		
Indirect-related	195	55–810	61	14–250	42	13–188		
Unrelated	4	0–102	1	0–30	1	0–20		
Diagnostic tests	3,006	2,134–4,511	1,536	1,152–2,332	701	536–1,199		
Direct-related	1,437	1,052–1,872	768	556–988	362	254–504		
Indirect-related	1,411	855–2,321	714	408–1,181	316	218–574		

Variable	Cumulative cost[#] Median	IQR	Out-of-pocket cost Median	IQR	Annual cost[¶] Median	IQR	Per visit/admission Mean	95% CI
Unrelated	6	0–69	4	0–37	1	0–16		
Clinical services	616	416–1,749	426	312–839	140	106–481		
Outpatient visits (n = 147)	Cumulative cost[#]		Out-of-pocket cost		Annual cost[¶]		Per visit[+]	
	Median	IQR	Median	IQR	Median	IQR	Median	IQR
Total	3,863	2,969–5,333	2,124	1,476–2,702	965	753–1,370	163	134–198
Non-benefit	35	7–115	35	7–115	8	2–32	1	0–5
Direct-related	1,978	1,521–2,652	976	704–1,266	514	374–738	89	60–111
Indirect-related	1,657	1,151–2,546	1,016	693–1,532	414	307–611	71	54–91
Unrelated	13	0–119	6	0–43	3	0–30	1	0–6
Medication	1,050	584–1,904	347	178–664	247	159–466	40	27–70
Direct-related	672	481–1,062	219	147–352	177	122–262	28	22–46
Indirect-related	121	19–647	40	6–200	28	5–136	4	1–22
Unrelated	0	0–56	0	0–17	0	0–13	0	0–2
Diagnostic tests	2,282	1,806–2,997	1,317	991–1,772	598	433–753	99	78–121
Direct-related	1,243	936–1,633	718	511–941	320	218–456	52	38–73
Indirect-related	1,006	609–1,431	556	296–849	250	160–324	41	28–52
Unrelated	0	0–35	0	0–18	0	0–8	0	0–1
Clinical services	448	347–597	368	272–474	110	87–137	19	17–20
Admission (n = 71)	Cumulative cost[#]		Out-of-pocket cost		Annual cost[¶]		Per admission[§]	
	Median	IQR	Median	IQR	Median	IQR	Median	IQR
Total	5,620	1,296–9,186	1,494	506–3,340	1,146	291–2,983	3,493	1,011–6,141
Non-benefit	326	107–887	326	107–887	65	24–224	212	103–452
Direct-related	496	152–1,571	142	26–336	94	33–357	291	125–881
Indirect-related	3,545	961–7,349	1,159	292–2,945	774	205–2,415	2,467	729–4,756
Unrelated	10	0–158	3	0–40	3	0–32	8	0–86
Medication	464	31–1,773	94	11–412	110	8–442	275	26–900
Direct-related	1	0–1,126	0	0–243	0	0–198	1	0–586
Indirect-related	120	30–468	30	7–120	24	7–127	72	22–272
Unrelated	0	0–28	0	0–7	0	0–10	0	0–19
Diagnostic tests	1,590	734–4,405	498	233–1,785	317	182–1,269	1,243	653–2,045
Direct-related	334	124–567	86	26–175	71	30–134	188	112–357
Indirect-related	1,016	429–3,535	338	153–1,210	218	103–925	740	395–1,505
Unrelated	0	0–96	0	0–25	0	0–18	0	0–63
Clinical services	1,470	289–4,118	348	72–1,000	359	66–1,382	1,111	200–2,634

*Currency in USD, rounded to the nearest dollar. IQR: Interquartile range.
#Includes all the costs pertaining to each category.
¶Cumulative cost divided by the duration of total follow-up in years.
+Cumulative cost for outpatient divided by the number of outpatient visits.
§ Cumulative cost of admission divided by the number of admissions.

Appendix Table 4. Comparison of costs between patients with and without admission*

Visits	Admission (n = 71)	Without Admission (n = 76)	p-value
All visits			
Total	9,429 [5,223–15,242]	3,773 [2,963–5,252]	<0.001
Medication	1,826 [955–3,944]	1,000 [520–1,715]	<0.001
Diagnostic tests	4,201 [3,065–6,833]	2,292 [1,823–2,937]	<0.001
Clinical services	1,794 [693–4,490]	435 [345–543]	<0.001
Outpatient visits			
Total	3,912 [3,004–5,361]	3,773 [2,963–5,252]	0.846
Medication	1,070 [617–2,237]	1,000 [520–1,715]	0.394
Diagnostic tests	2,184 [1,657–3,066]	2,292 [1,823–2,937]	0.551
Clinical services	490 [353–609]	435 [345–543]	0.158

*Data are represented as median [interquartile range]. Currency in USD, rounded to the nearest dollar.

Appendix Table 5. Cost analysis for the treatment of nontuberculous mycobacterial pulmonary disease, with patients with complete follow-up data*

	Cumulative cost#		Out-of-pocket cost		Annual cost¶		Per visit/admission	
Variable	Median	IQR	Median	IQR	Median	IQR	Mean	95% CI
All visits (n = 87)								
Total	5,470	3,613–9,914	2,592	1,714–4,112	1,284	845–2,268		
Medication	1,171	550–2,509	347	165–775	261	165–524		
Diagnostic tests	3,093	2,134–4,597	1,544	1,131–2,357	698	537–1,199		
Clinical services	633	435–1,749	479	306–865	140	107–394		
Outpatient visits (n = 87)	Cumulative cost#		Out-of-pocket cost		Annual cost¶		Per visit+	
	Median	IQR	Median	IQR	Median	IQR	Median	IQR
Total	3,736	2,946–5,162	2,020	1,402–2,615	939	663–1,351	162	132–189
Medication	983	524–1,627	320	157–592	219	146–402	37	27–62
Diagnostic tests	2,154	1,722–3,066	1,255	843–1,776	613	417–753	101	78–124
Clinical services	474	345–607	363	269–481	108	83–142	20	18–21
Admission (n = 45)	Cumulative cost#		Out-of-pocket cost		Annual cost¶		Per admission§	
	Median	IQR	Median	IQR	Median	IQR	Median	IQR
Total	4,519	1,124–9,051	1,686	367–3,435	805	224–2,994	968	968–6,904
Medication	242	27–1,388	55	7–337	42	6–285	21	21–915
Diagnostic tests	1,655	807–4,173	490	271–1,808	317	161–1,180	654	654–2,201
Clinical services	1,137	235–4,047	338	63–1,060	307	57–1,127	170	170–2,949

*Currency in USD, rounded to the nearest dollar. #: Includes all the cost pertaining to each category.
¶Cumulative cost divided by the duration of total follow-up in years.
+Cumulative cost for outpatient divided by the number of outpatient visits.
§Cumulative cost of admission divided by the number of admissions. IQR: Interquartile range.

Appendix Table 6. Baseline characteristics based on causative species*

Variable	MAC (n = 112)	MAB (n = 15)	p-value
Age at diagnosis, years	61.0 [54.0–67.8]	59.0 [46.0–64.0]	0.090
Sex, female	77 (68.8)	12 (80.0)	0.550
Comorbidities			
History of tuberculosis	18 (16.1)	4 (26.7)	0.293
History of NTM treatment	2 (1.8)	0 (0.0)	1.000
Bronchiectasis	28 (25.0)	5 (33.3)	0.535
Chronic obstructive lung disease	12 (10.7)	1 (6.7)	1.000
Asthma	6 (5.4)	3 (20.0)	0.073
Lung cancer	2 (1.8)	0 (0.0)	1.000
History of thoracic operation	10 (8.9)	1 (6.7)	1.000
Hypertension	6 (5.4)	0 (0.0)	1.000
Diabetes mellitus	4 (3.6)	0 (0.0)	1.000
Other malignancy	16 (14.3)	0 (0.0)	0.214
Duration, months			
Total duration of follow-up	51.4 [33.8–67.8]	33.1 [27.0–49.7]	0.006
Pre-diagnostic	0.2 [0.0–4.0]	0.2 [0.0–0.3]	0.135
Pre-treatment	3.8 [1.9–8.8]	2.6 [1.8–3.7]	0.150
Treatment	14.5 [13.3–18.3]	13.0 [12.8–15.2]	0.051
Post-treatment	19.8 [8.8–37.0]	17.0 [9.6–31.3]	0.424
Treatment duration >24 mo	9 (8.0)	0 (0.0)	0.598
Number of outpatient visits	22.0 [18.0–30.0]	32.0 [16.0–40.0]	0.110
Patients with admission	45 (40.2)	15 (100.0)	<0.001
Number of admissions	1.0 [1.0–2.0]	2.0 [1.0–3.0]	0.083
Length of stay per admission, days	3.0 [2.0–6.4]	20.7 [16.5–29.0]	<0.001
Total length of stay, days	5.0 [2.0–10.0]	33.0 [28.0–62.0]	<0.001
Treatment outcome			
Culture conversion	89 (79.5)	9 (60.0)	0.107
Microbiologic cure	75 (67.0)	9 (60.0)	0.576
Re-treatment for recurrence	9 (8.0)	2 (13.3)	0.618
All-cause mortality	4 (3.6)	1 (6.7)	0.472

*Data are presented as n (%) or median [interquartile range] unless indicated otherwise. NTM: nontuberculous mycobacteria; MAC: *M. avium* complex; MAB: *M. abscessus* .

Appendix Table 7. Expanded cost analysis based on causative species*

Variable	MAC Median	MAC Interquartile range	MAB Median	MAB Interquartile range	p-value
All visits		(n = 112)		(n = 15)	
Total	4,557	3,334–7,086	19,190	9,914–26,219	<0.001
Non-benefit	105	10–247	965	516–2,029	<0.001
Direct-related	2,118	1,638–2,740	3,869	3,061–5,622	<0.001
Indirect-related	2,044	1,336–3,725	13,227	6,339–18,754	<0.001
Unrelated	21	1–119	198	5–670	0.042
Medication	1,069	614–1,799	2,767	2,441–4,225	<0.001
Direct-related	640	487–1,055	2,459	1,792–3,326	<0.001
Indirect-related	168	45–589	461	112–898	0.049
Unrelated	2	0–57	41	0–346	0.043
Diagnostic tests	2,830	2,078–4,055	5,726	3,334–11,190	<0.001
Direct-related	1,416	1,051–1,804	1,483	1,149–2,297	0.331
Indirect-related	1,172	773–1,999	3,388	1,941–7,185	<0.001
Unrelated	5	0–47	39	0–326	0.206
Clinical services	541	377–930	6,093	4,263–11,972	<0.001
Outpatient visits		(n = 112)		(n = 15)	
Total	3,859	2,960–5,124	3,300	3,016–4,278	0.492
Non-benefit	23	6–107	215	13–390	0.003
Direct-related	1,998	1,515–2,631	1,714	1,542–2,325	0.331
Indirect-related	1,612	1,122–2,518	1,373	1,258–2,295	0.601
Unrelated	12	0–94	5	0–111	0.335
Medication	976	543–1,645	983	738–1,466	0.823
Direct-related	628	482–1,039	765	566–1,043	0.420
Indirect-related	111	20–501	98	19–429	0.659
Unrelated	0	0–48	0	0–90	0.344
Diagnostic tests	2,292	1,805–3,060	1,863	1,650–2,480	0.110
Direct-related	1,267	960–1,725	972	737–1,353	0.084
Indirect-related	992	580–1,455	872	562–1,303	0.339
Unrelated	0	0–28	0	0–5	0.195
Clinical services	444	347–564	506	318–643	0.399
Admission		(n = 45)		(n = 15)	
Total	1,710	933–7,747	14,197	6,711–22,521	<0.001
Non-benefit	247	103–882	562	340–1,782	0.032
Direct-related	240	97–518	2,152	1,569–3,107	<0.001
Indirect-related	1,191	675–6,368	10,932	4,968–15,945	<0.001
Unrelated	2	0–63	158	0–552	0.024
Medication	61	20–516	1,784	1,456–3,153	<0.001
Direct-related	0	0–2	1,579	1,126–2,431	<0.001
Indirect-related	53	20–462	272	99–537	0.034
Unrelated	0	0–4	31	0–198	0.004
Diagnostic tests	998	653–4,793	3,093	1,590–9,867	0.024
Direct-related	240	96–468	432	297–976	0.012
Indirect-related	698	407–3,563	2,067	1,223–6,312	0.018
Unrelated	0	0–35	23	0–298	0.236
Clinical services	556	147–1,863	5,639	3,847–11,439	<0.001

*Currency in USD, rounded to the nearest dollar. MAC, *M.avium* complex; MAB, *M. abscessus*.

Appendix Table 8. Cost analysis based on causative species, reported in mean and standard error*

Variable	*M.avium* complex Mean	*M.avium* complex 95% CI	*M.abscessus* Mean	*M.abscessus* 95% CI	p-value
All visits		(n = 112)		(n = 15)	
Total	6,339	3,604–9,075	20,260	12,784–27,735	0.001
Medication	1,665	1,049–2,281	4,224	2,540–5,907	0.017
Diagnostic tests	3,615	2,321–4,909	7,989	4,453–11,524	0.027
Clinical services	1,059	112–2,007	8,047	5,459–10,636	<0.001
Outpatient visits		(n = 112)		(n = 15)	
Total	4,426	3,967–4,886	3,960	2,704–5,217	0.480
Medication	1,464	1,174–1,754	1,365	571–2,158	0.009
Diagnostic tests	2,495	2,301–2,688	2,036	1,507–2,565	0.109
Clinical services	468	430–506	560	456–664	0.342
Admission		(n = 45)		(n = 15)	
Total	4,761	3,248–6,274	16,299	10,040–22,558	0.003
Medication	500	257–744	2,859	1,323–4,394	0.010
Diagnostic tests	2,788	1,931–3,646	5,953	2,658–9,248	0.087
Clinical services	1,473	868–2,077	7,487	5,094–9,881	<0.001

*Currency in USD, rounded to the nearest dollar. MAC, *M.avium* complex;. CI: confidence interval.

Appendix Table 9. Simple linear regression analysis of factors on cumulative cost*

Variable	B	Standard error	β	t	*p*-value	R^2
Age	75.429	101.421	0.062	0.744	0.458	
Sex (Female = 0, Male = 1)	3770.928	2602.739	0.119	1.449	0.150	
Comorbidities						
History of Tuberculosis	1852.811	3068.423	0.050	0.604	0.547	
History of NTM treatment	445.484	6744.734	0.005	0.066	0.947	
Bronchiectasis	71.822	2843.065	0.002	0.025	0.980	
COPD	202.091	3493.821	0.005	0.058	0.954	
Asthma	5036.257	4627.733	0.090	1.088	0.278	
Lung cancer	−2028.105	8645.224	−0.019	−0.235	0.815	
History of bronchiectasis, COPD, asthma or lung cancer	1349.158	2467.991	0.045	0.547	0.585	
Causative species (MAC = 0, MAB = 1)	13920.051	1768.805	0.576	7.870	<0.001	0.331
Total duration of follow-up (months)	23.251	50.235	0.038	0.463	0.644	
Treatment duration (months)	174.726	164.657	0.088	1.061	0.290	
Number of outpatient visits	455.609	60.041	0.533	7.588	<0.001	0.284
Number of admissions	10631.337	1391.750	0.677	7.639	<0.001	0.458
Any history of admission	9268.136	2322.390	0.315	3.991	<0.001	0.099
Total lengths of stay (days)	515.630	21.955	0.943	23.486	<0.001	0.889
Treatment outcomes						
Culture conversion	−3484.884	2948.456	−0.098	−1.182	0.239	
Microbiologic cure	−1803.964	2632.442	−0.057	−0.685	0.494	
Re-treatment for recurrence	4674.378	4288.491	0.090	1.090	0.278	
Death from any cause	34981.998	5453.435	0.470	6.415	<0.001	0.221

*NTM, non-tuberculous mycobacteria; COPD, chronic obstructive pulmonary disease; MAC, *M.avium* complex; MAB, *M.abscessus* .

Appendix Table 10. Baseline characteristics based on pulmonary comorbidities*

Variable	Comorbidities# present (n = 63)	No comorbidities# (n = 84)	p-value
Age at diagnosis, years	61.0 [56.0–69.0]	60.0 [52.3–66.0]	0.275
Sex, female	44 (69.8)	56 (66.7)	0.683
Other comorbidities			
History of tuberculosis	15 (23.8)	14 (16.7)	0.281
History of NTM treatment	3 (4.8)	2 (2.4)	0.652
History of thoracic operation	8 (12.7)	3 (3.6)	0.056
Hypertension	8 (12.7)	2 (2.4)	0.019
Diabetes mellitus	4 (6.3)	2 (2.4)	0.402
Other malignancy	9 (14.3)	8 (9.5)	0.372
Causative species			
M. avium complex	42 (66.7)	70 (83.3)	0.237
M. abscessus	9 (14.3)	6 (7.1)	
M. kansasii	6 (9.5)	4 (4.8)	
M. fortuitum	1 (1.6)	1 (1.2)	
Others¶	5 (7.9)	3 (3.6)	
Duration, months			
Total duration of follow-up	55.2 [34.3–74.4]	45.8 [31.6–63.8]	0.032
Pre-diagnostic	0.1 [0.0–2.2]	0.2 [0.0–0.7]	0.888
Pre-treatment	5.0 [2.5–9.8]	2.8 [1.6–6.0]	0.025
Treatment	15.3 [13.5–18.9]	14.1 [13.1–18.0]	0.056
Post-treatment	26.0 [10.7–38.5]	18.2 [8.2–34.6]	0.285
Treatment duration >24 mo	4 (6.3)	7 (8.3)	0.758
Number of outpatient visits	27.0 [20.0–36.0]	21.0 [17.0–32.0]	0.039
Patients with admission	36 (57.1)	35 (41.7)	0.063
Number of admissions	1.0 [1.0–2.0]	1.0 [1.0–2.0]	0.441
Length of stay per admission, days	7.5 [3.4–17.3]	4.5 [2.0–15.0]	0.116
Total length of stay, days	12.0 [5.0–30.8]	6.0 [2.0–20.0]	0.082
Treatment outcome			
Culture conversion	47 (74.6)	68 (81.0)	0.356
Microbiologic cure	41 (65.1)	60 (71.4)	0.411
Re-treatment for recurrence	4 (6.3)	9 (10.7)	0.397
All-cause mortality	2 (3.2)	4 (4.8)	0.701

*Data are presented as n (%) or median [interquartile range], unless otherwise indicated.
#Includes asthma, bronchiectasis, chronic obstructive pulmonary disease, and lung cancer.
¶Includes mixed infection. NTM: nontuberculous mycobacteria.

Appendix Table 11. Expanded cost analysis based on pulmonary comorbidities*

	Comorbidities[#] present		No comorbidities[#]		
Variable	Median	Interquartile range	Median	Interquartile range	p-value
All visits		(n = 63)		(n = 84)	
Total	6,999	4,556–12,312	4,182	3,260–7,837	<0.001
Non-benefit	134	26–732	107	9–323	0.071
Direct-related	2,505	1,817–3,545	2,252	1,706–2,812	0.198
Indirect-related	3,892	2,284–7,835	1,920	1,283–4,025	<0.001
Unrelated	42	0–319	27	1–125	0.483
Medication	2,129	939–3,944	1,069	543–1,803	<0.001
Direct-related	785	508–1,483	687	487–1,196	0.288
Indirect-related	508	168–1,564	101	29–275	<0.001
Unrelated	10	0–137	3	0–51	0.377
Diagnostic tests	3,629	2,372–4,918	2,524	2,010–3,761	0.005
Direct-related	1,488	1,060–2,053	1,410	1,041–1,779	0.393
Indirect-related	1,864	993–3,038	1,145	687–1,862	<0.001
Unrelated	5	0–94	12	0–55	0.921
Clinical services	761	479–3,407	528	369–1,062	0.002
Outpatient visits		(n = 63)		(n = 84)	
Total	4,454	3,203 - 6,429	3,558	2,628 - 4,670	0.004
Non-benefit	36	7 - 118	33	6 - 114	0.819
Direct-related	2,045	1,521 - 2,688	1,894	1,517 - 2,635	0.748
Indirect-related	2,211	1,411 - 3,289	1,449	983 - 2,288	<0.001
Unrelated	6	0 - 134	16	0 - 103	0.597
Medication	1,372	839 - 2,295	844	534 - 1,473	0.004
Direct-related	730	498 - 1,068	617	480 - 1,060	0.756
Indirect-related	327	66 - 1,240	69	14 - 211	<0.001
Unrelated	0	0 - 67	1	0 - 41	0.953
Diagnostic tests	2,446	1,874 - 3,333	2,124	1,694 - 2,874	0.047
Direct-related	1,246	905 - 1,790	1,235	960 - 1,614	0.731
Indirect-related	1,098	776 - 1,543	873	494 - 1,310	0.008
Unrelated	0	0 - 19	1	0 - 42	0.488
Clinical services	490	375 - 609	420	318 - 527	0.022
Admission		(n = 36)		(n = 35)	
Total	6,387	1,652–11,013	3,772	922–7,516	0.126
Non-benefit	394	105–1,013	317	107–599	0.557
Direct-related	554	131–1,740	369	179–993	0.404
Indirect-related	4,373	1,111–9,659	2,577	663–6,709	0.161
Unrelated	16	0–179	8	0–158	0.648
Medication	755	74–2,089	65	18–1,315	0.021
Direct-related	17	0–1,265	0	0–569	0.075
Indirect-related	184	67–524	53	18–463	0.008
Unrelated	0	0–31	1	0–27	0.903
Diagnostic tests	1,718	868–4,987	1,277	709–4,100	0.376
Direct-related	341	122–594	267	131–525	0.877
Indirect-related	1,232	484–4,136	834	404–3,010	0.295
Unrelated	0	0–102	8	0–86	0.478
Clinical services	2,354	508–4,672	1,104	200–3,948	0.139

*Currency in USD, rounded to the nearest dollar. [#]: Includes asthma, bronchiectasis, chronic obstructive pulmonary disease, and lung cancer.

Appendix Table 12. Cost analysis based on pulmonary comorbidities, reported in mean and standard error*

Variable	Comorbidities# present		No comorbidities#		p-value
	Mean	95% CI	Mean	95% CI	
All visits	(n = 63)		(n = 84)		
Total	9,762	6,114–13,410	8,413	5,254–11,572	0.585
Medication	2,675	1,853–3,496	1,946	1,235–2,658	0.190
Diagnostic tests	4,643	2,918–6,368	4,466	2,972–5,960	0.880
Clinical services	2,444	1,181–3,707	2,001	907–3,095	0.605
Outpatient visits	(n = 63)		(n = 84)		
Total	4,981	4,368–5,594	4,188	3,657–4,719	0.055
Medication	1,872	1,485–2,259	1,324	989–1,660	0.035
Diagnostic tests	2,609	2,351–2,867	2,393	2,170–2,617	0.217
Clinical services	500	449–551	470	426–514	0.380
Admission	(n = 36)		(n = 35)		
Total	8,367	5,364–11,370	10,141	1,485–18,796	0.702
Medication	1,404	670–2,138	1,493	−97–3,082	0.921
Diagnostic tests	3,560	2,294–4,826	4,974	729–9,219	0.529
Clinical services	3,403	2,102–4,703	3,674	733–6,616	0.868

*Currency in USD, rounded to the nearest dollar. #: Includes asthma, bronchiectasis, chronic obstructive pulmonary disease, and lung cancer. CI: confidence interval.

Appendix Table 13. Analysis of out-of-pocket costs based on causative species*

Variable	*M.avium* complex		*M.abscessus*		p-value
	Median [IQR]	% of cumulative cost	Median [IQR]	% of cumulative cost	
All visits	(n = 112)		(n = 15)		
Total	2,328 [1,730 - 3,174]	51%	5,765 [3,891 - 14,670]	30%	<0.001
Medication	321 [183 - 598]	30%	972 [752 - 1,344]	35%	<0.001
Diagnostic tests	1,464 [1,108 - 1,998]	52%	2,611 [1,544 - 3,830]	46%	<0.001
Clinical services	385 [307 - 583]	71%	1,603 [1,351 - 4,788]	26%	<0.001
Outpatient visits	(n = 112)		(n = 15)		
Total	2,045 [1,456 - 2,645]	53%	2,138 [1,681 - 2,757]	65%	0.665
Medication	314 [165 - 524]	32%	547 [349 - 676]	56%	0.014
Diagnostic tests	1,302 [979 - 1,767]	57%	1,119 [993 - 1,506]	60%	0.354
Clinical services	358 [273 - 457]	81%	454 [272 - 552]	90%	0.167
Admission	(n = 45)		(n = 15)		
Total	619 [312 - 2,949]	36%	3,568 [1,753 - 10,044]	25%	<0.001
Medication	13 [5 - 152]	21%	433 [317 - 700]	24%	<0.001
Diagnostic tests	388 [206 - 1,784]	39%	1,066 [440 - 2,834]	34%	0.027
Clinical services	126 [43 - 647]	23%	1,222 [968 - 4,515]	22%	<0.001

*Currency in USD, rounded to the nearest dollar. *P*-values for the comparison of the median values. IQR, interquartile range.

Appendix Table 14. Analysis of out-of-pocket costs based on pulmonary comorbidities*

Variable	Comorbidities# present		No comorbidities#		p-value
	Median [IQR]	% of cumulative cost	Median [IQR]	% of cumulative cost	
All visits	(n = 63)		(n = 84)		
Total	3,156 [2,293 - 5,126]	45%	2,209 [1,578 - 3,152]	53%	<0.001
Medication	642 [282 - 1,175]	30%	321 [163 - 625]	30%	<0.001
Diagnostic tests	1,894 [1,239 - 2,602]	52%	1,378 [1,103 - 1,896]	55%	0.008
Clinical services	583 [376 - 1,319]	77%	374 [288 - 577]	71%	<0.001
Outpatient visits	(n = 63)		(n = 84)		
Total	2,381 [1,726 - 2,941]	53%	1,896 [1,429 - 2,526]	53%	0.005
Medication	506 [263 - 766]	37%	291 [160 - 475]	34%	0.002
Diagnostic tests	1,423 [1,088 - 1,918]	58%	1,240 [916 - 1,567]	58%	0.058
Clinical services	419 [308 - 531]	86%	334 [254 - 441]	80%	0.008
Admission	(n = 36)		(n = 35)		
Total	1,886 [621 - 3,707]	30%	749 [331 - 3,016]	20%	0.118
Medication	203 [13 - 498]	27%	13 [4 - 317]	20%	0.048
Diagnostic tests	540 [302 - 1,797]	31%	440 [209 - 1,415]	34%	0.441
Clinical services	737 [114 - 1,219]	31%	215 [46 - 901]	19%	0.072

*Currency in USD, rounded to the nearest dollar. *P*-value for the comparison of the median values. #: Include asthma, bronchiectasis, chronic obstructive pulmonary disease, and lung cancer. IQR, interquartile range.

Appendix Table 15. Cost of surgical management in nontuberculous mycobacterial pulmonary disease treatment, for five patients who received surgical treatment*

Variable	Median	Interquartile range
Total cost	19,190	14,264–94,780
Surgical costs only	9,077	8,566–19,866
Total days of admission for surgery, days	37.0	5.5–129.0
Total cost of admission for surgery	17,192	9,084–70,562
Cost including perioperative care	17,277	9,629–70,822

*Currency in USD, rounded to the nearest dollar.

Appendix Table 16. Cost of management for complications related to the treatment of nontuberculous mycobacterial pulmonary disease*

		Department-specific cost		Total cost	
Patients with visits to	n (%)[#]	Median	IQR	Median	IQR
Any non-pulmonology	81 (55.1)	573	187–2,515	7,840	4,400–13,596
Otorhinolaryngology	28 (19.0)	72	61–158	13,329	10,056–22,821
Ophthalmology	21 (14.3)	147	135–178	4,883	3,677–15,927

*Currency in USD, rounded to the nearest dollar. [#]: Out of 147 patients. IQR: Interquartile range.

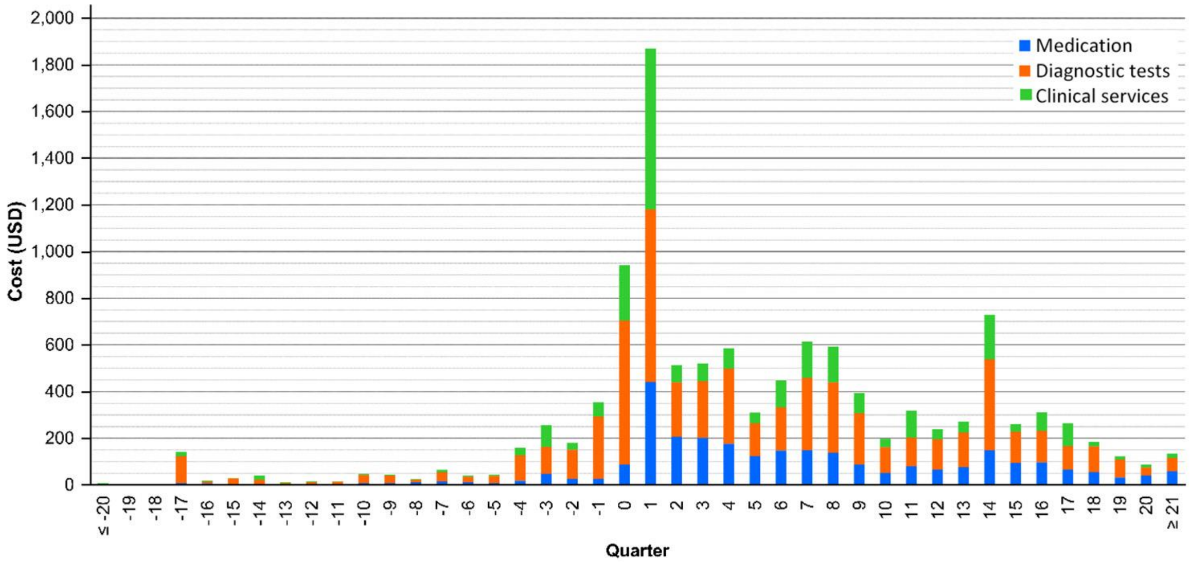

Appendix Figure 1. Trend of average quarterly costs by category. A stacked histogram of average quarterly costs for each category (in USD) is plotted with respect to time from treatment initiation. Quarter 1 denotes the first quarter after treatment initiation.

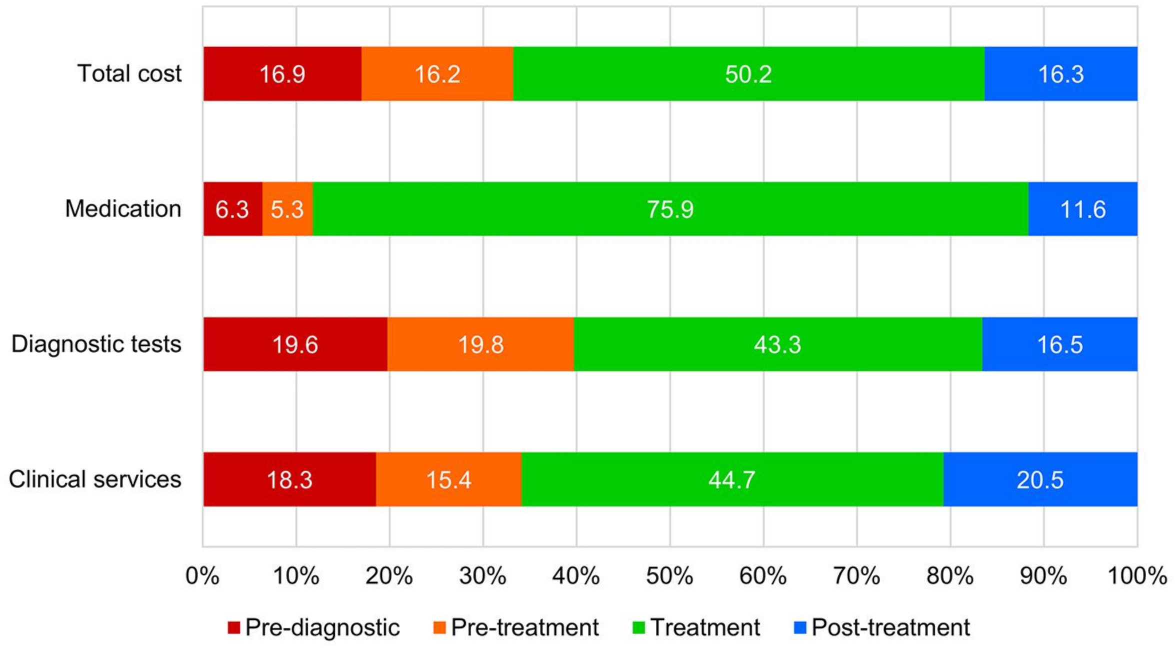

Appendix Figure 2. Distributions of costs based on each follow-up segment. The cost distributions for each follow-up segment were calculated. Data for patients involving cost data for all the four follow-up segments (n = 87) are presented.
